# Medical nutrition therapy during the first 5 days of critical illness: a retrospective cohort study

**DOI:** 10.1590/1806-9282.20260939

**Published:** 2026-10-30

**Authors:** Aranza Michelle Sánchez Vázquez, Lilia Castillo-Martínez, Eduardo Rivero-Sigarroa, José Ángel Baltazar-Torres, María Fernanda Bernal-Ceballos, José Luis Villanueva-Juárez

**Affiliations:** 1 Servicio de Nutriología Clínica del Instituto Nacional de Ciencias Médicas y Nutrición Salvador Zubirán, Unidad de Terapia Intensiva – Mexico City, Mexico.; 2 Servicios Públicos de Salud del Instituto Mexicano del Seguro Social para el Bienestar, Unidad de Atención a la Salud – Mexico City, Mexico.

**Keywords:** Enteral nutrition, Parenteral nutrition, Critical illness, Shock, Vasoconstrictor agents, Nutrition therapy

## Abstract

**OBJECTIVE:**

The aim of this study was to describe the prescription of medical nutrition therapy for critically ill patients who were admitted to an intensive care unit during the first 5 days.

**METHODS:**

In an observational, longitudinal, retrospective study conducted at the intensive care unit of a tertiary hospital in Mexico City, adult patients admitted from January to December 2018 and from November 2024 to February 2025 were included. Indications for fasting, the route of medical nutrition therapy (enteral nutrition—parenteral nutrition), caloric and protein intakes, sequential organ failure assessment score and lactate levels, and vasopressor requirements during the 5 days were evaluated. Delay in initiating medical nutrition therapy was defined as initiated after 48 h of intensive care unit admission.

**RESULTS:**

100 critically ill patients were included; aged 53±18.8, and with a median sequential organ failure assessment score of 10 (7–12). The most frequent diagnoses at admission were pulmonary disease and septic shock. On the first day, 98% of patients were fasting, and 25% received high-dose vasopressor therapy (if the norepinephrine equivalent dose >0.3 μg/kg/min). Over 5 days, medical nutrition therapy transitioned progressively, predominantly from fasting through enteral nutrition (45%) and parenteral nutrition (11%). medical nutrition therapy delay in 54 patients and in a multivariate analysis was associated with age (OR 1.04; 95%CI 1.01–1.07), sequential organ failure assessment score (OR 1.20; 95%CI 1.02–1.40), and lactate levels (OR 1.8; 95%CI 1.10–3.00).

**CONCLUSION:**

Prescription of medical nutrition therapy in critically ill patients admitted to a intensive care unit during 5 days is dynamic, and in all patients, it is not possible to initiate at 48 h, associated with higher age and illness severity.

## INTRODUCTION

Critically ill patients frequently present with hemodynamic instability, and fluid resuscitation is the immediate therapeutic priority. This condition is accompanied by intense inflammatory and metabolic responses that induce catabolic stress and adversely affect nutritional status. Therefore, medical nutrition therapy (MNT), delivered via enteral nutrition (EN) or parenteral nutrition (PN), is considered a fundamental intervention, and its timely initiation is recommended in clinical practice^1,2^.

Hemodynamic instability can lead to organ dysfunction or death within minutes to hours. The initial priorities are fluid resuscitation, airway management, and adequate oxygen supply^1^. Additionally, administering vasoactive substances—vasopressors and inotropes—is essential to restore hemodynamic stability and prevent further organ deterioration^3^. However, the gastrointestinal system is the first to be affected during hemodynamic instability with reduced blood flow and oxygenation, resulting in ischemia and hypoxia at the tips of the intestinal villi. This phenomenon can persist even after systemic blood flow is restored^3,4^. Simultaneously, the metabolic response to stress is characterized by the redistribution of energetic and structural substrates, including hypermetabolism, hyperglycemia, hyperlactatemia, accelerated protein catabolism, and a hyperdynamic cardiovascular state^5,6^. This process involves neuroendocrine, inflammatory, immunological, and hormonal activation, with the release of cytokines that promote anorexia, proteolysis, and lipolysis^7-9^.

Critical illness is associated with a progressive loss of lean body mass and persistent hypermetabolism, with marked deterioration in nutritional status^10,11^. In this context, MNT plays an important role, as there is consensus that adequate MNT can mitigate the consequences of catabolism^2^. International clinical nutrition guidelines^12,13^ recommend initiating MNT within 48 h, with EN when the digestive tract is fully or partially functional—either early or late^14,15^, permissive^16^ or trophic^17^—and initiating complementary or total PN when the gastrointestinal tract is insufficient or non-functional^12^.

However, evidence regarding the prescription of MNT in routine medical practice for critically ill patients is divergent, including strategies for personalization according to severity status, clinic, vasoactive drug doses, and timing of initiation^2,18,19^. This study aimed to describe the prescription of MNT during the first 5 days for critically ill patients admitted to an intensive care unit (ICU).

## PATIENTS AND METHODS

### Design and study population

A retrospective cohort study was conducted on critically ill adult patients admitted to the ICU of a tertiary National Health Institute between January 2018 and December 2018 and between November 2024 and February 2025. The COVID-19 pandemic period was excluded because medical-nutrition therapy differed during that period. Enrollment was consecutive. The study was approved by the Ethics and Research Committee (Ref: TEI-2656-18-19-1) and conducted in accordance with the Declaration of Helsinki. Patients >18 years old, with a diagnosis of critical illness [presence of acute organ dysfunction evidenced by sequential organ failure assessment (SOFA) score ≥2 requiring advanced organ support, either invasive mechanical ventilation, renal replacement, or vasopressor support] and data from the first 5 days of stay in the ICU available in the electronic clinical record were included. Patients who stayed in the ICU for less than 24 h, those who died within the first 24 h of admission to the ICU, and those who received MNT prior to admission with incomplete data on vasopressor use or MNT indications were excluded.

Demographic data, diagnosis at admission, SOFA score, lactate and phosphorus levels, and 24-h urine output, as well as pharmacological therapy including norepinephrine and vasopressin prescription with average daily doses, were recorded at ICU admission and during the early hours of each day over a 5-day stay. Hemodynamic instability was defined as the need for an infusion of at least one vasopressor agent. Norepinephrine equivalent dose (NEQ) was calculated as follows^20^:

NEQ=[norepinephrine (μg/kg/min)]+[dopamine (μg/kg/min)/150]+[adrenaline (μg/kg/min)]+[phenylephrine (μg/kg/min)/10]+[vasopressin (unit/min)/0.4].

High-dose vasopressor therapy was considered if the NEQ dose >0.3 μg/kg/min.

Total energy expenditure was estimated based on the patient's ideal weight, with a goal of 100% of requirements. For EN and PN, the goal was 25 kcal/kg/day and 1.5 g of protein/kg/day. The ideal weight was calculated using the following World Health Organization formula^21^: Men: Ideal weight=Height (m)^2^×23; Women: Ideal weight=Height (m)^2^×21.5.

Energy and protein adequacy were calculated as the percentage of the established goal contributed. For EN, we recorded the administered volume, formula caloric density, and protein intake. For PN, glucose and amino acid levels, as well as intravenous lipid emulsion, were quantified to determine daily caloric and amino acid intake.

Fasting was defined as the absence of oral, enteral, and parenteral feeding. MNT delay was defined as patients for whom medical nutrition therapy (MNT) was initiated after 48 h of intensive care unit (ICU) admission.

## Statistical analysis

Comparisons between groups with or without MNT delay on categorical variables were performed using the chi-square test. For comparisons of continuous variables between groups, Student's t-test or the Mann‒Whitney U test was used. Multivariate analysis to identify variables associated with MNT delay was performed using logistic regression; Akaike's criterion was used to select variables for inclusion in the model. Statistical analyses were performed using Jamovi version 2.6.44.0. Statistical significance was set at p<0.05.

Nominal variables related to MNT indication and vasopressor use over the 5-day study period were analyzed and ­visualized using alluvial diagrams.

## RESULTS

During the study period, 320 patients were admitted to the ICU. Of these, 142 remained hospitalized for at least 5 days and were screened. A total of 22 patients were transferred to a non-critical care area, and 20 were excluded because of incomplete information on vasopressor use or MNT indication. Therefore, 100 patients were included in the analysis.

Table 1 presents the baseline characteristics and pharmacological prescription therapy of the study population on day 1. Overall, the mean age was 53±18.8, and 57% of patients were female. The most frequent admission diagnoses were pulmonary disease and septic distributive shock.

**Table 1:** Baseline demographic and clinical characteristics of the study population and comparison between patients with delay in medical nutrition therapy.

Variables		Total n=100	Delay MNT n=54	Not delay MNT n=36	p*
	Age, years	53±18.8	55.1±18.5	48±17	0.08
	Sex, female/male, n (%)	57 (57)/43 (43)	26 (48)/28 (52)	20 (55.6)/16 (44.4)	0.65
Main reason for ICU admission, n (%)					
	Pulmonary disease	36 (36)	13 (24.1)	22 (61.1)	0.001
	Septic and distributive shock	9 (9)	12 (22.2)	1 (3.2)	
	Autoimmune disease	11 (10.2)	5 (9.3)	5 (13.9)	
	Cardiovascular disease	11 (10.2)	6 (11.1)	1 (2.7)	
	Gastrointestinal disorders	10 (9.3)	6 (11.1)	2 (5.5)	
	Transplantation	10 (9.3)	5 (9.3)	0	
	Other	13 (12.2)	7 (13)	5 (13.9)	
Clinical condition					
	SOFA score at admission	10 (7–12)	12 (9–13)	8 (6–11.5)	0.01
	Invasive mechanical ventilation, n (%)	74 (74)	38 (70.4)	33 (91.7)	0.02
Biochemical variables at admission					
	Lactate (mmol/L)	2 (1.2–3.1)	2.3 (1.7–4.0)	1.6 (1.1–3.5)	0.003
	Phosphorus (mg/dL)	4.3±1.8	4.6±1.9	4.3±2.2	0.57
	Urine 24-h output (mL)	1,360 (793–2,319)	1,185 (627–1,761)	1,659 (985–2,635)	0.90
Pharmacological therapy day 1					
	Norepinephrine, n (%)	63 (63)	40 (74)	22 (61.1)	0.16
	Doses (μg/kg/min)	0.3 (0.27–0.49)	0.3 (0.26–0.46)	0.3 (0.25–0.57)	0.11
	Vasopressin, n (%)	22 (22)	22 (40.7)	4 (11.1)	0.003
	Doses (units/min)	0.04 (0.03–0.06)	0.05 (0.03–0.06)	0.04 (0.03–0.06)	0.60
	NEQ (μg/kg/min)	0.27±0.19	0.31±0.18	0.24±0.19	0.16
	Antibiotic, n (%)	67 (67)	34 (63)	28 (77.8)	0.08
Opioid, n (%)					
	Tramadol	10 (9.3)	0 (0)	2 (5.6)	0.09
	Fentanyl	65 (65)	37 (65.8)	28 (77.8)	0.09
Sedative, n (%)					
	Propofol	26 (26)	20 (40.7)	14 (38.9)	0.90
	Midazolam	19 (19)	9 (16.7)	10 (27.7)	0.21

MNT: medical nutrition therapy; ICU: intensive care unit; SOFA: Sequential Organ Failure Assessment; NEQ: norepinephrine equivalent. Values are expressed as n (%), mean±SD, or median (p. 25–75), as appropriate.

* Comparisons between groups were performed using the χ^2^ test, t-test, or Mann-Whitney U test, depending on the nature of the variable.

Among excluded patients, 59% were female, and the mean age was 48.4±16.5 years; transplantation was the most frequent admission diagnosis. Compared with included patients, excluded patients did not differ significantly by sex (p=0.85) or age (p=0.15).

On the first day, 98% of patients were fasting, and 25% were receiving high-dose vasopressor therapy. Across the 5-day observation period, MNT transitions followed a progressive pattern, moving mainly from fasting on day 1 through EN (45%) and PN (11%) on day 5. The proportion of patients receiving oral feeding increased progressively (Figure 1), whereas use of high-dose vasopressor therapy decreased.

**Figure 1:**
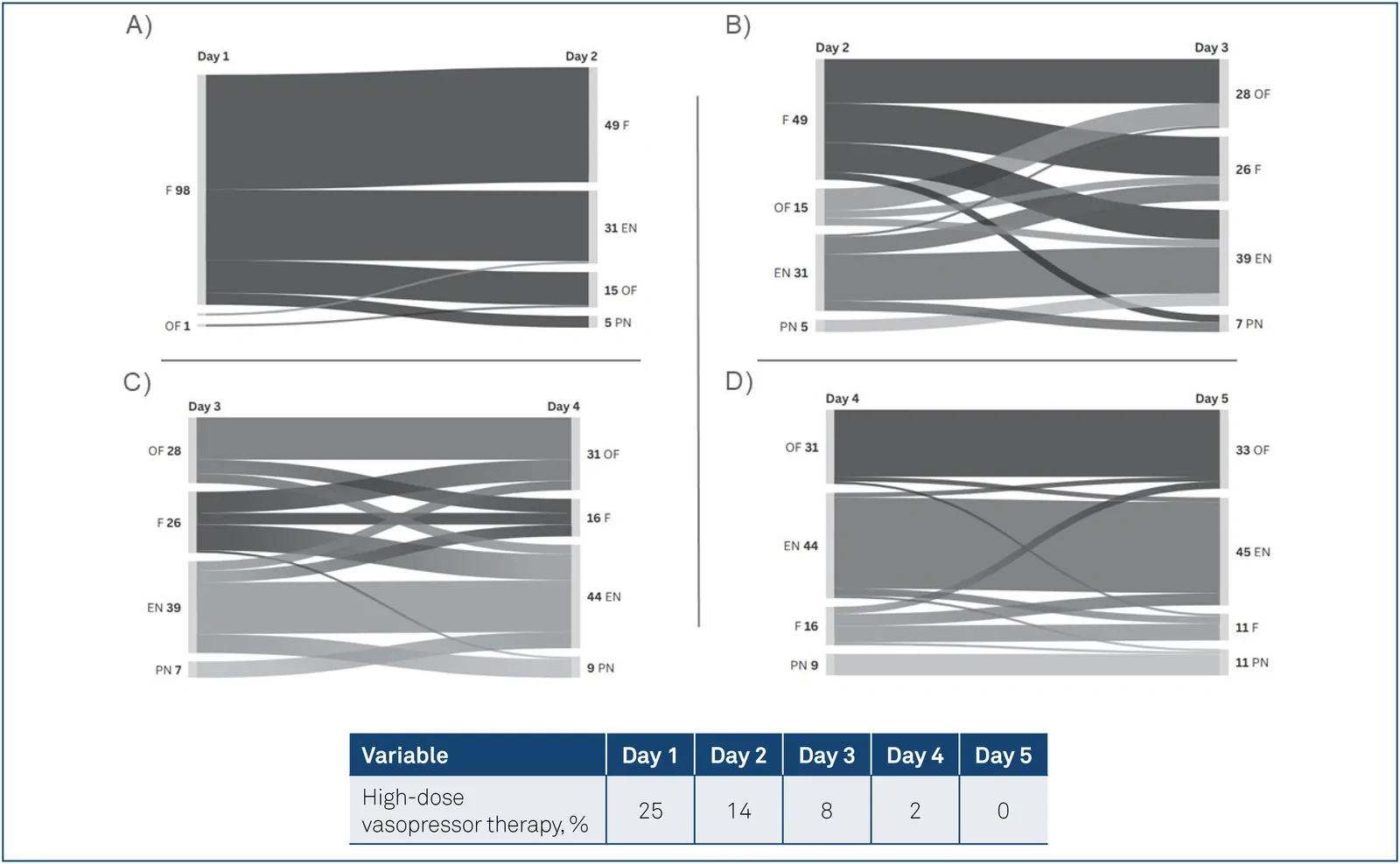
MNT trajectory during the first 5 days of intensive care unit stays. Data are presented as a percentage of patients with fasting, enteral nutrition, parenteral nutrition, and oral feeding. (A) Dynamic transition between day 1 and day 2, see https://public.flourish.studio/visualisation/30020325/; (B) dynamic transition between day 2 and day 3, see https://public.flourish.studio/visualisation/30020665/; (C) dynamic transition between day 3 and day 4, see https://public.flourish.studio/visualisation/30020521/; (D) dynamic transition between day 4 and day 5, see https://public.flourish.studio/visualisation/30021153.

Among the 36 patients who received MNT, 86.1% received EN, and 13.9% received PN within 48 h. Median energy delivery was 13 (7–23.5) kcal/kg/day, and median protein delivery was 0.99 (0.4–1.4) g/kg/day.

Estimated average energy requirements were 1,613 kcal/day for EN and 2,050 kcal/day for PN, with protein intake goals of 78.4 g/day and 98.4 g/day, respectively. EN goals were not achieved on any of the 5 days; by day 5, caloric adequacy was 51.1%, and protein adequacy was 45%. Caloric adequacy for PN increased, reaching 99.7% on day 5, while protein adequacy reached 76.3%.

MNT initiation was delayed in 54 patients and was not delayed in 36. When clinical characteristics were compared between these groups (Table 1), the delayed group had older age, a higher proportion of septic distributive shock as the main reason for ICU admission, higher SOFA scores, higher lactate levels, more frequent vasopressin therapy, and a lower requirement for invasive mechanical ventilation. These differences were statistically significant, except for age.

In the multivariate analysis, delayed MNT initiation was associated with age, SOFA score, and lactate levels (Table 2).

**Table 2:** Logistic regression variables associated with delay in medical nutrition therapy.

Variables	OR	95%CI	p
Age (years)	1.04	1.01–1.07	0.021
SOFA score	1.20	1.02–1.40	0.029
Lactate (mmol/L)	1.8	1.10–3.00	0.020

SOFA: Sequential Organ Failure Assessment; OR: odds ratio; CI: confidence interval.

## DISCUSSION

In this study of critically ill patients hospitalized for at least 5 days, delayed MNT initiation was common, occurring mainly in patients with greater illness severity. More than half of patients had delayed MNT initiation, and only 36 received MNT within the first 48 h. In multivariate analysis, age, SOFA score, and lactate levels remained independently associated with delayed MNT initiation, suggesting that baseline vulnerability and acute physiologic instability influenced decisions to postpone nutritional support.

This likely reflects the acute context of early critical illness, hemodynamic instability, postoperative status, prioritization of resuscitation, and intensive monitoring. In this setting, fasting may be involuntary because of impaired consciousness or disease severity or medically indicated as a total, partial, or intermittent therapeutic measure during clinical or surgical procedures, depending on the patient's condition^22^.

Distributive shock was the most frequent cause of hemodynamic instability. Its initial management prioritized fluid therapy to restore circulating volume and vasoactive drugs to re-establish homeostasis^3^. On day 1, 25% of patients required infusion vasopressor therapy, indicating substantial hemodynamic compromise. Adverse effects require consideration. The splanchnic circulation, vulnerable to vasoconstriction during high-dose vasopressor therapy. Reduced intestinal blood flow may cause mucosal hypoxia and ischemia, impair barrier function, and limit nutrient absorption. Under these conditions, enteral MNT could increase splanchnic oxygen demand and worsen ischemia, supporting fasting during the acute phase of hemodynamic instability^3,14,23^. Mancl et al. reported that lower vasopressor requirements were associated with greater tolerance and indication for EN^24^. Lactate was associated with delayed MNT initiation. It is a bedside marker of tissue hypoperfusion and resuscitation adequacy; elevated values may delay nutritional advancement due to concern for splanchnic hypoperfusion and intolerance. Nevertheless, it should be interpreted within the broader clinical trajectory, suggesting it guided nutrition decisions in this ICU population. Improving lactate with stable vasopressors at doses <0.3 μg/kg/min may support trophic EN.

The independent association between SOFA score and delayed MNT initiation indicates, as previously stated, that organ dysfunction influenced timing of nutritional support, likely requiring more intensive resuscitation and monitoring^19^.

Critical illness triggers a metabolic stress response, and acutely, exogenous nutrient delivery may exceed the patient's metabolic capacity, producing a positive energy balance that increases oxygen demand and aggravates oxidative stress^6,25^. This pathophysiological context supports a permissive or trophic MNT strategy during phases of critical illness. As vasopressor requirements decreased, fasting became less frequent and EN prescription increased, suggesting hemodynamic stability was key to MNT initiation. Nevertheless, despite MNT coverage, caloric and protein targets were not achieved during the 5-day period among patients receiving EN, consistent with evidence in critically ill populations^2^. MNT prescription should be individualized through assessment of evolution, including hemodynamic stability, organ and gastrointestinal function, and gasometric, electrolyte, and metabolic parameters, to guide timing, route, and dose of MNT.

The assumption that nutritional intake counteracts catabolism and improves prognosis remains debated. Evidence, including findings reported by Hartl et al., suggests metabolic adaptation during critical illness may be more effective when aggressive nutritional support is avoided in initial phases^22^. Therefore, MNT should support organ function and adaptation while allowing recovery. Once vasoactive support is no longer required, MNT goals should be reassessed and redirected toward optimizing nutritional status according to condition.

This study has limitations due to its observational and retrospective design, which precludes establishing causal relationships between vasopressor use and MNT prescription. Similarly, confounding variables, such as clinical severity and variability in clinical management, could have affected results. Hemodynamic stability was evaluated using clinical parameters and vasopressor use, which did not fully reflect tissue perfusion. Additionally, as this was a single-center study, the generalizability of the findings is limited.

In conclusion, the prescription of MNT in critically ill patients admitted to a ICU during 5 days is dynamic, and in all patients it is not possible to initiate at 48 h, associated with higher age and illness severity.

## Data Availability

The datasets generated and/or analyzed during the current study are available from the corresponding author upon reasonable request.
